# Analysis of Microbial Community Structure in the Coastal Waters of Island Cities: Based on High-Throughput Sequencing and Traditional Bacterial Culturing

**DOI:** 10.7759/cureus.106512

**Published:** 2026-04-06

**Authors:** Pengwei Hou, Dan Ye, Ziqi Li, Zihuan Zeng, Shousen Wang, Liangfeng Wei

**Affiliations:** 1 Department of Neurosurgery, Fuzong Clinical Medical College of Fujian Medical University, Fuzhou, CHN; 2 Department of Neurosurgery, Oriental Hospital Affiliated to Xiamen University, Fuzhou, CHN; 3 Department of Neurosurgery, 900th Hospital of the Joint Logistics Support Force of the People's Liberation Army of China (900 Hospital), Fuzhou, CHN

**Keywords:** illumina miseq sequencing, marine bacteria, pathogen, seawater, vibrio

## Abstract

Background: Coastal water microbial communities are highly diverse and often undergo changes with variations in environmental conditions. This study focuses on the seawater around Pingtan, utilizing Illumina MiSeq sequencing (San Diego, California, United States) and bacterial cultivation techniques to analyze the abundance and relative composition of planktonic bacterial communities.

Methods: On May 26, 2023, seawater samples were collected at Liushui Port, Guanyin'ao Port, and Qianbian'ao Port in Pingtan County. At each sampling point, random samples were taken at 500 meters, 1000 meters, and 1500 meters, and relevant physicochemical factors were measured. Samples were processed using a suction filtration system to obtain nine filters, grouped into O1, O2, and O3 based on the offshore distance of the sampling points. Subsequently, total seawater bacterial sequencing was conducted. The remaining seawater samples were centrifuged for bacterial cultivation and identification. Alpha-diversity indices, including species coverage, species diversity indices (Shannon-Wiener and Simpson indices), richness estimates (Chao1), and abundance-based richness estimates (ACE), were calculated to assess the diversity of planktonic bacteria in seawater. Beta-diversity was employed to evaluate differences between samples. Statistical analysis was performed using the R software (R Foundation for Statistical Computing, Vienna, Austria).

Results: The mean α-diversity indices for the O1 group were 345 OTUs, Chao 420, Ace 403.12, Shannon index 3.34, and Simpson index 0.11. In the O2 group, the values were 451 OTUs, Chao 494.08, Ace 491.46, Shannon index 3.34, and Simpson index 0.07. The O3 group exhibited values of 430 OTUs, Chao 467.99, Ace 465.40, Shannon index 3.51, and Simpson index 0.09. At the phylum level, in all groups, *Proteobacteria* were the most abundant phylum (55.32-77.74%). In the O1, O2, and O3 groups, the dominant genus was *Vibrio*, constituting 16.64%, 8.12%, and 13.77%, respectively. A total of 15 different bacterial species, comprising 57 strains, were successfully cultured from seawater bacterial cultivation. The findings suggest significant differences between the bacterial communities of O1 and O2 in ocean bacterioplankton.

Conclusions: The marine bacterial community exhibits a high abundance of bacterial taxa, including *Proteobacteria*, *Cyanobacteria*, and *Bacteroidetes*. The most prevalent genera are *Vibrio*, *Pseudoalteromonas*, and *Candidatus*_*Pelagibacter*. Importantly, the composition of bacteria varies with different nearshore distances.

## Introduction

The ocean spans more than 70% of the Earth's surface and contains an enormous diversity of microorganisms [1]. Marine microbial communities are extraordinarily complex and play essential ecological roles. It has been estimated that the ocean contains approximately 1×10^29^ bacterial cells and these microorganisms have undergone more than 3.5 billion years of evolution [2]. Among marine microbes, bacteria and archaea are the most abundant groups, comprising both autotrophic primary producers and heterotrophic taxa involved in the turnover of dissolved organic carbon and nutrients [3,4]. Nevertheless, the composition of bacterial communities differs markedly among marine environments [4], and community structure is continually reshaped by environmental fluctuations. Previous studies have demonstrated that salinity, temperature, oxygen concentration, water depth, nutrient availability, and geographic setting all influence marine bacterial diversity [5].

A worldwide analysis of 77,000 marine sequences showed that 57% were unique at a 98% identity threshold, highlighting the high heterogeneity of marine ecosystems. Along the Mediterranean coastline, *Proteobacteria* (69%) and *Bacteroidetes *(27%) are the predominant bacterial phyla, whereas *Cyanobacteria *comprise only 1% of the community [6]. In the western English Channel, *Proteobacteria *constitute the dominant phylum, accounting for 50.3% of the total bacterial assemblage [7]. In the Bohai Sea, the northern Yellow Sea, and the North Atlantic Ocean, *Proteobacteria *and *Bacteroidetes *are likewise the major phyla, representing 77% and 18.2% of the communities, respectively [8,9].

Although the majority of marine bacteria are nonpathogenic, certain species are capable of causing infections in humans [10,11]. These pathogens may invade the body through the ingestion of contaminated seafood or through skin wounds, potentially resulting in severe illness or death. In the United States, around 8,000 cases of *Vibrio*-related disease are reported each year [12]. Some marine bacteria can infect humans and animals through wounds and rapidly progress to serious conditions such as sepsis, as documented in earlier reports [13,14]. Therefore, a clearer understanding of marine bacterial community composition may contribute to improved diagnosis and management of infections caused by these organisms.

The waters around Pingtan Island lie on the continental shelf of the East China Sea and serve as a connection between the South China Sea and the East China Sea. This area is recognized as a representative upwelling zone [15]. In summer, seawater from the South China Sea enters the waters surrounding Pingtan Island, leading to elevated temperature and salinity. These hydrographic changes affect phytoplankton growth and subsequently alter the abundance of plankton, mollusks, and fish in aquaculture zones along the southeastern coast of China [16,17]. Moreover, harmful algal blooms caused by species such as *Karenia mikimotoi *and *Akashiwo sanguinea *occur frequently in the Pingtan region and substantially influence the structure of marine bacterial communities [18]. For this reason, investigation of microbial community structure in this area is of considerable significance.

Since most marine microorganisms cannot be cultivated under laboratory conditions [19], molecular approaches have become important tools for microbial community research. Advances in high-throughput technologies, particularly high-throughput sequencing, have enabled the more comprehensive characterization of marine bacterial communities. High-throughput sequencing has clear advantages in terms of efficiency, cost, and accuracy of information. Among currently available platforms, Illumina MiSeq (San Diego, California, United States) offers relatively low sequencing cost, high accuracy, and a short experimental turnaround, making it especially suitable for amplicon sequencing. This technology provides an effective approach for a more detailed investigation of marine microorganisms [20].

The present study aimed to (1) characterize the physicochemical properties of the seawater environment along the coast of Pingtan and (2) investigate the composition of the total bacterial community and active bacterial populations at the phylum, genus, and species levels by integrating Illumina MiSeq sequencing with conventional bacterial culture methods.

## Materials and methods

Seawater collection and measurement of marine environmental factors 

Seawater samples were collected from three coastal sites in Pingtan County, Fujian Province, China, namely, Liushui Port, Guanyin'ao Port, and Qianbian'ao Port, on May 26, 2023. These three sites were selected to represent different coastal locations within the Pingtan area. At each site, samples were obtained at approximately 500 m, 1000 m, and 1500 m from the shore to evaluate potential changes in bacterial community composition along an offshore-distance gradient. The nine samples were subsequently grouped according to offshore distance as O1 (500 m), O2 (1000 m), and O3 (1500 m).

Seawater samples were collected from approximately 50 cm below the surface. Physicochemical parameters, including water temperature, pH, dissolved oxygen, salinity, turbidity, and related environmental variables, were measured in situ during sampling using a multiparameter water quality detector (JY-500D, China). The instrument was calibrated according to the manufacturer's instructions before field sampling.

**Figure 1:**
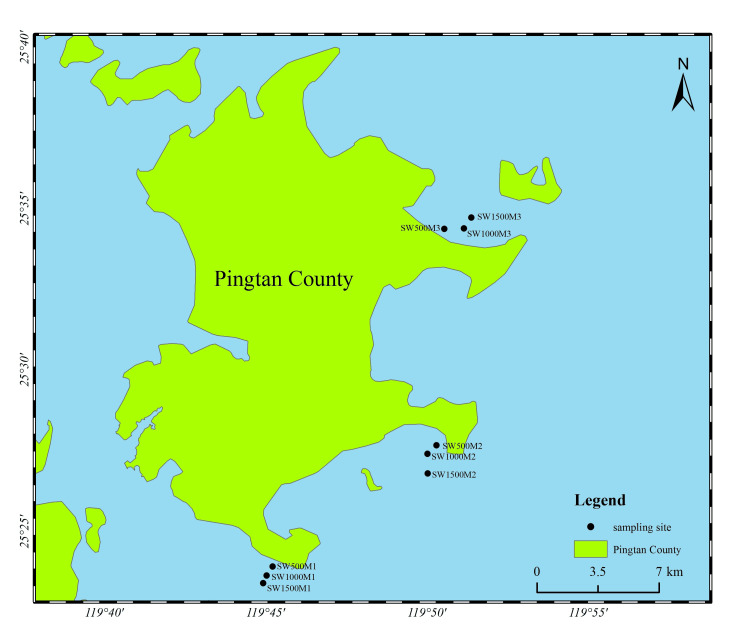
Map of the sampling stations at Pingtan County, Fujian Province, China Sampling locations in the coastal waters of Pingtan County, Fujian Province, China. Black dots indicate the nine sampling stations located 500 m, 1000 m, and 1500 m from shore at three coastal sites. The figure was created by the authors using ArcGIS (Environmental Systems Research Institute, Inc. (Esri), Redlands, California, United States).

For microbial community analysis, each seawater sample was processed using a suction filtration system, and 1500 mL of seawater was filtered through a 0.22 μm membrane filter with a diameter of 50 mm. Three filter membranes were prepared for each offshore-distance group, yielding a total of nine membranes containing bacterial biomass, designated SW500M1, SW1000M1, SW1500M1, SW500M2, SW1000M2, SW1500M2, SW500M3, SW1000M3, and SW1500M3 (Figure 1).

Bacterial culture and identification

After centrifugation of O1 (SW500M1, SW500M2, SW500M3), O2 (SW1000M1, SW1000M2, SW1000M3), and O3 (SW1500M1, SW1500M2, SW1500M3), the supernatant was decanted, and a 1 mL aliquot of the sediment was aseptically transferred into 25 mL of alkaline peptone water using a sterile 2 mL syringe. The mixture was subsequently incubated at 35°C for 6-8 hours to promote resuscitation of sublethally injured bacterial cells. After incubation, the samples were used to inoculate thiosulfate-citrate-bile salts-sucrose agar (TCBS) and blood agar plates, followed by incubation at 35°C for 18-24 hours. In parallel, an additional 1 mL of the bottom fluid was mixed with 25 mL of *Shigella *enrichment broth and incubated at 35°C for 18-24 hours. Surface cultures were then streaked onto *Salmonella Shigella* (SS) agar and xylose lysine deoxycholate (XLD) agar to detect any pathogenic colonies, followed by incubation at 35°C for 18-24 hours. Bacterial colonies were then identified using an automated microbial identification system (VITEK® MS, bioMérieux, Lyon, France) based on matrix-assisted laser desorption/ionization time-of-flight mass spectrometry (MALDI-TOF MS) and compared to references from a proprietary database.

DNA extraction and 16S rRNA gene amplification with polymerase chain reaction (PCR)

The total community genomic DNA was extracted with an E.Z.N.A™ MagBind Soil DNA Kit (Omega Bio-tek, Norcross, Georgia, United States), according to the manufacturer's instructions. We measured the concentrations of the DNA using a Qubit™ 4.0 fluorometer (Thermo Fisher Scientific, Waltham, Massachusetts, United States) to ensure that adequate amounts of high-quality genomic DNA had been extracted. The DNA fragments in all samples ranged from 500 bp to 1500 bp. The quality test results are all qualified. Our target was the V3-V4 hypervariable region of the bacterial 16S rRNA gene. PCR was performed immediately after the DNA was extracted. The 16S rRNA V3-V4 amplicon was amplified with 2×Hieff® Robust PCR Master Mix (Yeasen, 10105ES03, Shanghai, China). Two universal bacterial 16S rRNA-gene-specific PCR primers (purified with polyacrylamide gel electrophoresis)were used: forward primer 5¢-CCTACGGGNGGCWGCAG-3¢ and reverse primer 5¢-GACTACHVGGGTATCTAATCC-3¢. The reaction (30 µL) contained 2 µL of microbial DNA (10 ng/µL), 1 µL of forward primer (10 µM), 1 µL of reverse primer (10 µM), and 2×Hieff® Robust PCR Master Mix (Yeasen). The PCR was performed in the Applied Biosystems 9700 thermocycler (15 µL) (ETC 811, Beijing, China) with the following cycling parameters: one cycle of denaturation at 95°C for three minutes; five cycles of denaturation at 95°C for 30 seconds, annealing at 45°C for 30 seconds, and elongation at 72°C for 30 seconds; 20 cycles of denaturation at 95°C for 30 seconds, annealing at 55°C for 30 seconds, and elongation at 72°C for 30 seconds; and a final extension at 72°C for five minutes. The PCR products were checked with electrophoresis on 2% (w/v) agarose gels in Tris-boric acid-EDTA (TBE) buffer stained with ethidium bromide and visualized under UV light.

16S gene library construction, quantification, and sequencing

We used Hieff NGS™ DNA selection beads (Yeasen, 10105ES03) to remove any free primers and primer dimer species from the PCR products. The samples were delivered to Sangon Biotech (Shanghai, China) for library construction with Illumina TruSeq. Before sequencing, the DNA concentration of each PCR product was determined with a Qubit® 4.0 green double-stranded DNA assay, and the DNA quality was confirmed with an Agilent 2100 bioanalyzer (Agilent, Santa Clara, California, United States). Depending on the coverage required, all the libraries were pooled for a single run. The amplicons from each reaction mixture were pooled in equimolar amounts based on their concentrations. Sequencing was performed with the Illumina MiSeq system, according to the manufacturer's instructions.

Sequence processing, operational taxonomic unit (OTU) clustering, representative tag alignment, and biological classification 

After sequencing, paired-end reads were assembled based on overlap between the two short-read sets using the PEAR software (Version 0.9.8, The Exelixis Lab, Heidelberg Institute for Theoretical Studies, Heidelberg, Germany). FASTQ files were processed to generate separate FASTA and QUAL files and then analyzed using standard procedures. Effective tags were clustered into OTUs at a similarity threshold of ≥97% using the USEARCH software (Version 11.0.667, Edgar R, Lawrence Berkeley National Lab (LBNL), Berkeley, California, United States).

Chimeric sequences and singleton OTUs, defined as OTUs represented by only one read, were removed. The remaining sequences were assigned to samples according to OTU classification. The most abundant tag sequence within each cluster was selected as the representative sequence. Representative bacterial OTU sequences were taxonomically assigned by BLAST searches against the Ribosomal Database Project (RDP) database.

Statistical analysis

Alpha-diversity indices, including the Chao1, Simpson, and Shannon indices, were used to quantify OTU richness and diversity. Rarefaction curves based on observed OTUs were constructed to assess sampling adequacy, and all alpha-diversity indices were calculated using the Mothur software (Version 1.39.5, Schloss Lab, University of Michigan, Ann Arbor, Michigan, United States).

To estimate microbial community diversity within samples, Student's t-test was used for comparisons between two groups, whereas one-way analysis of variance (ANOVA) was used for comparisons among multiple groups. Correlation coefficients and p-values among communities or OTUs were calculated using SparCC (Version 1.1.0, Friedman and Alm Lab, Massachusetts Institute of Technology, Cambridge, Massachusetts, United States).

In addition, exploratory Spearman correlation analyses were performed to assess the associations between physicochemical parameters and microbial diversity indices. Because of the limited sample size, these analyses were considered exploratory and interpreted with caution.

## Results

Analysis of the diversity and richness of OTUs

As shown in Table 1, in group O1, 73,448 v3-v4 16S rRNA hypervariable region sequences, ranging from 19,582 to 61,226 nucleotides (nt) in length, were obtained with MiSeq sequencing. In the O2 group, 107,570 v3-v4 sequences, ranging from 28,262 to 46164 nt, were obtained. In group O3, 83,622 v3-v4 16S rRNA sequences, ranging from 22,604 to 33,140 nt, were obtained. After random resampling with a difference threshold of 3%, the mean α-diversity indices of the O1 group were 345 OTUs, Chao 420, Ace 403.12, Shannon index 3.34, and Simpson index 0.11 (Table 1), those for the O2 group were 451 OTUs, Chao 494.08, Ace 491.46, Shannon index 3.34, and Simpson index 0.07, and those for the O3 group were 430 OTUs, Chao 467.99, Ace 465.40, Shannon index 3.51, and Simpson index 0.09.

**Table 1:** Alpha-diversity indices of different groups (such as Number, OTUs, Shannon, Chao, Ace, Simpson, Shannoneven, and Coverage)

Sample	Number	OTUs	Shannon	Chao	Ace	Simpson	Shannoneven	Coverage
SW500M1	24186	390	3.07	467.34	449.96	0.15	0.51	1
SW500M2	29680	420	3.27	507.13	492.72	0.11	0.54	1
SW500M3	25582	225	3.66	288.08	266.67	0.06	0.68	1
SW1000M1	42164	470	3.61	502.90	502.40	0.09	0.59	1
SW1000M2	31744	454	3.66	515.29	506.09	0.08	0.60	1
SW1000M3	28262	430	4.14	464.044	465.89	0.04	0.68	1
SW1500M1	27878	415	3.48	443.5	448.63	0.11	0.58	1
SW1500M2	22604	427	3.58	479.36	475.26	0.10	0.59	1
SW1500M3	33140	448	3.89	481.11	472.32	0.07	0.64	1

Association between environmental variables and microbial diversity

To further explore the potential influence of environmental factors on microbial diversity, exploratory Spearman correlation analyses were performed between major physicochemical variables and alpha-diversity indices. The results suggested that variation in water temperature, dissolved oxygen, turbidity, and salinity may be associated with differences in microbial diversity across sampling groups. However, given the limited sample size, these findings should be interpreted cautiously (Table 2).

**Table 2:** Environmental factors among different groups during seawater collection

Environmental factors	Group O3	Group O2	Group O1
Temperature (℃)	28.0	28.0	28.0
Water temperature (℃)	24.30	25	25.60
pH	7.68	7.66	7.66
Dissolved oxygen	9.76	9.11	7.12
Turbidity (NTU)	14.0	14.3	16.4
Wind speed in the Beaufort scale (0-12)	4-5	3-4	0-1
Salinity (‰)	28.8	28.9	28.1
Offshore distance (m)	1500±100	1000±100	500±100

Phylum level

**Figure 2:**
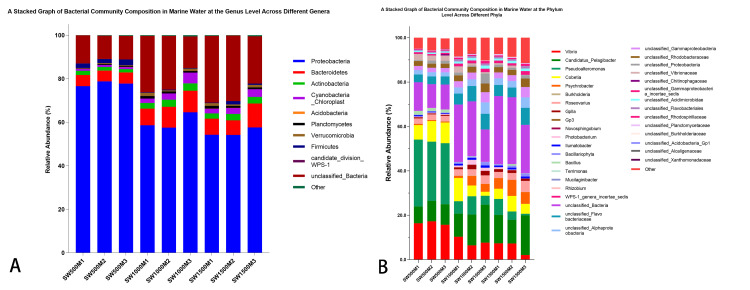
Bacterial community composition (A) Stacked graph of bacterial community composition in marine water at the phylum level across different phyla. The proportion of SW500M deformed bacteria in Group B (77.74%) is significantly higher than in other groups. (B) Stacked graph of bacterial community composition in marine water at the genus level across different genera. *Pseudoalteromonas *and *Vibrio *are dominant bacterial taxa in group SW500M, while the bulk of sequences in groups SW1000M and SW1500M are associated with unclassified bacteria, whose taxonomic identification falls below the predefined threshold level of credibility. Such a discrepancy may also arise due to the incomplete database of amplified sequences obtained from the sample in question.

The bacterial community of the O1 group was the most abundant of the three communities at the phylum level (Figure 2A), and the three most abundant phyla were *Proteobacteria *(77.74%), *Bacteroidetes *(5.05%), and *Acidobacteria *(1.69%). In the bacterial community of the O2 group, the three most abundant phyla were *Proteobacteria *(60.22%), *Bacteroidetes *(8.87%), and *Cyanobacteria *(9.05%). In the bacterial community of the O3 group, the three most abundant phyla were *Proteobacteria *(55.32%), *Bacteroidetes *(8.34%), and *Acidobacteria *(2.85%) (Figure 2A).

Genus level

The three most abundant marine bacterial genera in the O1 group were *Pseudoalteromonas *(28.78%), *Vibrio *(16.64%), and *Candidatus_Pelagibacter *(8.73%). The three most abundant marine bacterial genera in the O2 group were *Candidatus_Pelagibacter *(13.80%), *Vibrio *(8.12%), and *Cobetia* (5.71%). The three most abundant marine bacterial genera in the O3 group were *Candidatus Liberibacter* spp. (13.77%), *Psychrobacter *(5.77%), and *Vibrio *(5.56%) (Figure 2B).

Bacterial culture and identification

Three sampling points yielded 53 bacterial strains from seawater. O1, O2, and O3 yielded 23, 17, and 13 bacterial strains, respectively. *Vibrio *species, including *Vibrio alginolyticus *and *Vibrio parahaemolyticus*, were the dominant species of the family *Vibrionaceae*, while *Escherichia coli *and *Enterococcus *species were the major species of other families of bacteria (Table 3).

**Table 3:** Bacterial strains isolated from seawater at different sampling groups Data are presented as n.

Bacteria		O1, n	O2, n	O3, n	Total, n
Vibrio alginolyticus		3	3	3	9
Vibrio parahaemolyticus		2	3	3	8
Vibrio cholerae		2	2	3	7
Vibrio vulnificus		1	2	1	4
Vibrio metschnikovii		0	0	1	1
Escherichia coli		3	3	1	7
Klebsiella pneumoniae		3	2	0	5
Enterococcus faecalis		3	2	1	6
Acinetobacter baumannii		1	0	0	1
Pseudomonas aeruginosa		2	0	0	2
Staphylococcus saprophyticus		1	0	0	1
Staphylococcus epidermidis		1	0	0	1
Staphylococcus aureus		1	0	0	1
Total	23		17	13	53

Sample comparative analysis

**Figure 3:**
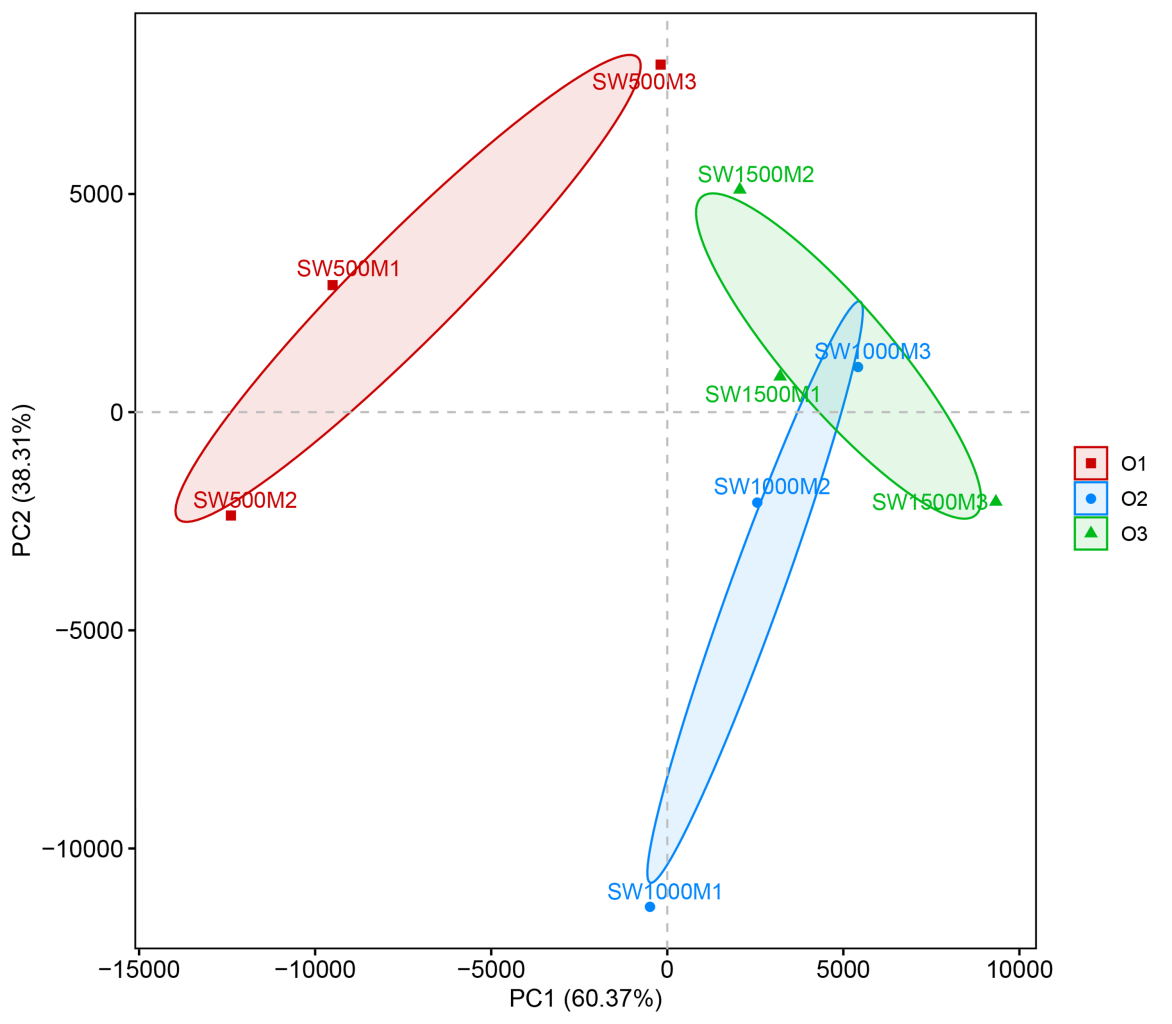
PCoA of bacterial communities in the O1, O2, and O3 groups based on unweighted UniFrac distances The figure illustrates the statistical analysis of structural and compositional differences among O1, O2, and O3 groups using PCoA based on unweighted UniFrac distance matrix in beta-diversity analysis. Inter-group differences were analyzed through ANOSIM, revealing a significant separation into three clusters for O1, O2, and O3 groups (R=1; p<0.05). PCoA: principal coordinates analysis; ANOSIM: analysis of similarities; UniFrac: Unique Fraction

Beta-diversity analysis based on unweighted Unique Fraction (UniFrac) distances revealed clear separation among the O1, O2, and O3 groups in the principal coordinates analysis (PCoA) plot (Figure 3). PC1 and PC2 explained 60.37% and 38.31% of the overall variation, respectively. Analysis of similarities (ANOSIM) further demonstrated significant differences among the three groups (R=1; p<0.05). The O1 samples formed a distinct cluster that was clearly separated from O2 and O3, indicating marked differences in bacterial community structure. In addition, within the O1 group, sample SW500M3 showed a relatively distinct position compared with the other two O1 samples.

## Discussion

To the best of our knowledge, this is the first study to integrate Illumina MiSeq sequencing with conventional culture techniques to investigate both the physicochemical characteristics and bacterial community structure of coastal seawater in the Pingtan region. Illumina MiSeq analysis demonstrated that the bacterial community was mainly composed of *Proteobacteria*, *Cyanobacteria*, *Bacteroidetes*, and *Actinobacteria *at the phylum level, whereas *Pseudoalteromonas*, *Vibrio*, and *Marinobacter *were the predominant genera. Moreover, bacterial community composition changed with offshore distance, and these differences were particularly evident among taxa with relatively low abundance.

Dominant community composition revealed by Illumina MiSeq sequencing

The major bacterial groups identified in this study were broadly in agreement with previous investigations of marine bacterial communities from different parts of the world [21-23]. In our samples, *Proteobacteria *represented the most abundant phylum, which contrasts with observations from some other coastal environments, including Florida and Chennai, where *Cyanobacteria *were found to predominate [24,25]. In the present dataset, *Cyanobacteria *accounted for 9.05% of the overall bacterial community. *Proteobacteria *constitute an extremely diverse phylum, encompassing both ubiquitous environmental microorganisms and clinically relevant pathogens. Members of this phylum exhibit substantial morphological, physiological, and ecological heterogeneity and include bacteria of major significance to human and animal health, such as *Vibrio *spp. and *Escherichia coli *[26]. In the current study, *Vibrio *was one of the dominant genera identified in the study area, in line with the results reported by Wang et al. [27].

Dominant cultivable bacterial species and their characteristics

At the species level, culture-based analysis identified *Vibrio parahaemolyticus*, *Vibrio vulnificus*, *Vibrio alginolyticus*, *Escherichia coli*, *Enterococcus faecalis*, and *Klebsiella pneumoniae *as the major cultivable bacteria. Notably, *Vibrio*-related species were dominant among the cultured isolates, which is broadly consistent with the sequencing results showing that *Vibrio *was one of the dominant genera in the study area. However, the cultured profile did not completely match the sequencing-based community structure, likely because culture-based methods recover only a subset of bacteria that can grow under the selected laboratory conditions, whereas Illumina MiSeq-based sequencing provides a more comprehensive view of the natural microbial community [25].

Bacterial composition in seawater at different offshore distances

Bacterial proliferation and community organization are closely associated with environmental conditions. Seawater temperature, salinity, dissolved oxygen, and other physicochemical variables can all shape bacterial community composition [28]. In the present study, these physicochemical characteristics differed across sampling sites at varying offshore distances. Specifically, seawater temperature declined with increasing distance from shore, whereas dissolved oxygen and turbidity showed an increasing trend. These environmental gradients may partly account for the observed differences in bacterial community composition among the sampling groups. Duan et al. suggested that varying environmental drivers, together with spatial and temporal habitat constraints, may result in niche differentiation within marine planktonic communities [29]. These exploratory results suggest that environmental gradients may partly contribute to the observed differences in microbial community composition across offshore distances, although larger studies with broader temporal and spatial coverage are needed to confirm these associations.

Based on the culture results, the number of cultivable bacterial isolates decreased as the offshore distance increased. This pattern may be related to environmental changes across the sampling gradient, and marine plastic debris may also contribute to this trend [30].

Limitations

Several limitations of this study should be acknowledged. First, although the sampling sites and offshore distances varied, all samples were obtained from the same city, which may limit the broader applicability of the findings. Future research should include a wider geographic range to better characterize regional variation in marine bacterial communities. Second, 16S rRNA gene amplicon sequencing has limited taxonomic resolution for certain organisms, especially at the species level. As a result, species-level conclusions derived solely from sequencing data should be interpreted with caution. Even so, genus-level community profiles still offer meaningful ecological insights and may indirectly assist in inferring dominant species. Third, culture-based identification is restricted by the selectivity and sensitivity of cultivation conditions, whereas high-throughput sequencing can partially overcome these shortcomings by providing a more inclusive picture of the microbial community.

Overall, the combined use of conventional culture methods and Illumina MiSeq sequencing offered complementary insights into the bacterial composition of coastal seawater in the Pingtan region and may contribute to the assessment of the potential public health relevance of marine bacterial communities.

## Conclusions

This study employed Illumina MiSeq sequencing in conjunction with bacterial cultivation techniques to conduct an in-depth investigation into the composition of microbial communities in the coastal waters of Pingtan. Bacterial taxa exhibiting high abundance in the marine bacterial community included *Proteobacteria*, *Cyanobacteria*, and *Bacteroidetes*. The most prevalent genera were *Vibrio*, *Pseudoalteromonas*, and *Candidatus_Pelagibacter*. Importantly, bacterial composition varied with different offshore distances. Exploring the differences in composition and distribution of marine bacteria not only serves as a cautionary measure for individuals engaged in nearshore activities but also holds significant implications for the diagnosis and treatment of bacterial infections related to seawater.
